# Comparative study of the usability of two software programs for visualization and analysis of digital orthodontic models

**DOI:** 10.15171/joddd.2018.033

**Published:** 2018-09-18

**Authors:** Matheus Felter, Milena Moraes de Oliveira Lenza, Maurício Guilherme Lenza, Wendel Minoro Muniz Shibazaki, Rhonan Ferreira Silva

**Affiliations:** ^1^School of Dentistry, Federal University of Goias, Goiânia, Goias, Brazil; ^2^Orthodontist, Master in Dentistry, Goiânia, Goias, Brazil; ^3^School of Dentistry, Estadual Paulista University, Araraquara, São Paulo, Brazil

**Keywords:** Dental models, dental technology, orthodontics

## Abstract

***Background.*** Software programs for visualization and analysis of digital orthodontic models, apart from presenting the necessary features for diagnosis and treatment planning, also need to be user-friendly. This characteristic refers to software’ usability, a measure that evaluates how easy it is to use it is by a specific group of professionals. The aim of this study was to compare the usability of free available versions of two software programs for visualization and analysis of digital orthodontic models.

***Methods.*** Digimodel® and OrthoCAD® usability were evaluated through their interface analysis and executing the following procedures: malocclusion classification and models analysis (arch-length and tooth-size discrepancies).

***Results.*** Digimodel® and OrthoCAD® software programs had an installer only for Windows platform, occupied less than 110 megabytes of virtual space and only read files from their respective manufacturers. None possessed Portuguese as a language option. Both allowed visualization of the models in different axes through options present in initial screen, at a click. For model analysis, both software programs required to measure tooth to tooth and performed necessary calculations automatically. However, OrthoCAD® software program was less intuitive because the option for these actions was among several others, within menus, which could cause confusion during navigation. In addition, the marking of points did not always obey the clicked site.

***Conclusion.*** The free access version of the evaluated software programs exhibited usability limitations related to language, supported file format and even the model analysis execution for orthodontic diagnosis. Although OrthoCAD® was inferior, both did not meet orthodontists’ clinical demand against these factors in the evaluated versions.

## Introduction

The representative models of dental arches were introduced to dentistry in the early 1700's and their possibility of use was improved with the development of the technique and the materials used in their preparation.^1^ In orthodontics, plaster models have been used for more than 100 years for diagnostic and treatment planning purposes,^2^ and are analyzed by orthodontists to identify and classify malocclusions,^3^ assess arch-length discrepancie,^4,5^ verify the compatibility of dental volume between the arches,^6^ plan and simulate mechanics indicated for dental movements in the arches (setups)^7^ and check the need for possible dental slices.^8^ Commonly, during or at the end of treatment, they are also used as reference for fabricating appliances such as orthodontic retainers, using acrylic resin.^9^

In addition to the clinical importance, plaster models can also be used as evidence in judicial proceedings initiated by patients,^10^ in cases of human identification,^11^ in the communication process between professionals and in the education and research sectors.^1^

At the end of the 1990s, digital models were introduced to orthodontics, facilitating characteristics in relation to physical plaster models, such as digital storage, speed gain and automation of dental units and interdental space measurements, and the possibility of simulation of several treatment options in the same pair of models.^12^

The transition from plaster to digital in orthodontics, according to the literature,^13,14^ is a coming reality. However, some authors^15-17^ consider the high cost and learning curve related to the interaction of the user with software interface as disadvantageous for their clinical use.

The use of a software program for visualization and analysis of orthodontic digital models is acceptable, like any diagnostic method, when it presents high reliability;^18,19^ i.e., it produces accurate results and with good agreement when applied at different moments by the same^20^ or by different examiners.^21^ Studies point to the existence of accuracy and reliability in the analysis of digital models in relation to plaster models.^22,23^ However, even if it possesses all the necessary features for the models analysis,^24^ even for clinicians who possess sufficient skill and knowledge, the software program must be accessible in terms of costs^25,26^ and exhibit good usability.

According to ISONORM 9241, which deals with the interaction between people and machines, usability is a measure that evaluates the ease with which a specific group of professionals can understand and perform a certain task in a computer program.^27^ Westerlund et al^28^ evaluated four software programs in orthodontics and verified that all had deficiencies in this measure, needing improvements to make the technology better and widely used by orthodontists.

Inaccurate measures taken by orthodontists for diagnostics, whether due to inability, lack of knowledge at some stage of dental treatment, or even limitations in their basic training,^29^ can generate clinical, ethical and legal questioning of their function,^30^ as the number of litigations against orthodontists have been increasing.^31^

In this sense, it becomes important that free software programs available for the orthodontist to visualize and analyze digital models meet usability criteria, supplying their clinical demand for diagnosis and case planning.

The aim of the present study was to compare the usability of free available versions of two software programs for visualization and analysis of digital orthodontic models (Digimodel® and OrthoCAD®).

## Methods

First, software programs to be used in this paper were selected. For this purpose, a research was carried out in the literature, through Pubmed database (www.pubmed.gov.br), between 2007 and 2017, for works in which software prograsms had been used to compare measurements made in digital models with those taken in plaster models. Of 75 articles found, those with another objective were excluded. Finally, there were 16 software names in 33 papers (Table 1).

**Table 1:** Software programs found in scientific literature vs. number of papers in which they were utilized

**Software program**	**Appearances**
OrthoAnalyzer®	8
Digimodel®	4
O3DM®	3
Rapidform®	3
BibliocastCecile3®	2
Emodel®	2
OraMetrix®	2
OrthoCAD®	2
AnatoModels®	1
Ivoris@Analyze3D®	1
MatLab®	1
Meshlab®	1
O3D®	1
Ortho3D®	1
OrthoInsight®	1
Pixform®	1

The software programs were selected based on the following criteria: a) number of appearances in the literature and b) availability of a free version to download on the manufacturer's website. This way, Digimodel® (OrthoProof, New Mexico, USA) and OrthoCAD® (Align Technology, San Jose, California, USA) software programs were selected for comparison because, based on the two established criteria, these were the first ones matching them. However, with fewer appearances than other software programs, at the time of this survey (August 2017), OrthoCAD® was the software program, found by researchers, as the second option that provided a free version available for use.

The two selected software programs were then downloaded to a personal computer, along with sample models provided by the manufacturers and analyzed separately. Software usability analysis was performed by checking, mainly, the following items:

- Type of installer
- Virtual space required to install the program
- File formats supported by the program
- Languages available for navigation
- Possibility of classifying malocclusion in digital models
- Possibility of performing model analysis (arch-length and tooth-size discrepancies)

Data were annotated and the interfaces were compared and discussed according to possible advantages or disadvantages related to them.

## Results

Digimodel® and OrthoCAD® software programs had an installer only for Windows platform, occupied less than 110 megabytes of virtual space each and only read files from their respective manufacturers. Both presented icons as action buttons to assist in the users’ orientation for a more intuitive navigation, and none of them possessed Portuguese as a language option.

Only OrthoCAD® allowed saving measurements or tasks that were performed in a session of use of the program to continue later. In addition, it also allowed the export of images of the work screen in JPEG format, unlike Digimodel®, which had export options in HTML and TXT format, but blocked in the evaluated version (Table 2).

**Table 2:** Software programs’ technical information

	**Digimodel®**	**OrthoCAD®**
**CHARACTERISTICS**		
Installer platform	Windows	Windows
Virtual space	22 *megabytes*	107 *megabytes*
Supported file format	Manufacturer’s only (.opds)	Manufacturer’s only (.3dm)
Language	English	English/Russian
Available features	- Marking of points to perform measurements (option available on the software’s initial screen, described as "measurements"); - Visualization of the upper and lower models, separated or occluded, in different views, by means of a click, in the software initial screen; - Possibility to visualize models in order to establish malocclusion classification; - Automatic calculation of overjet and overbite in two clicks (no need of marking points for these tasks); - Individual tooth-length measurements and annotations (3 clicks: 1 for tooth selection, and 2 for marking points); - Automatic calculations of arch-length and tooth-size discrepancies (it is needed that teeth dimensions are already marked).	- Marking of points to perform measurements (option available after opening a “Diagnostic” menu and clicking the “measurements” option within it); - Visualization of the upper and lower models, separated or occluded, in different views, by means of a click, in the software initial screen; - Possibility to visualize models in order to establish malocclusion classification; - Automatic calculation of overjet and overbite in two clicks (no need of marking points for these tasks); - Individual tooth-length measurements and annotations (3 clicks: 1 for tooth selection, and 2 for marking points); - Automatic calculation of arch-length and tooth-size discrepancies (it is needed that teeth dimensions are already marked); - Automatic calculations for Korkhaus analysis; - Possibility to save the work done to continue later; - Possibility to export screenshots in JPEG format.

**Figure 1:**
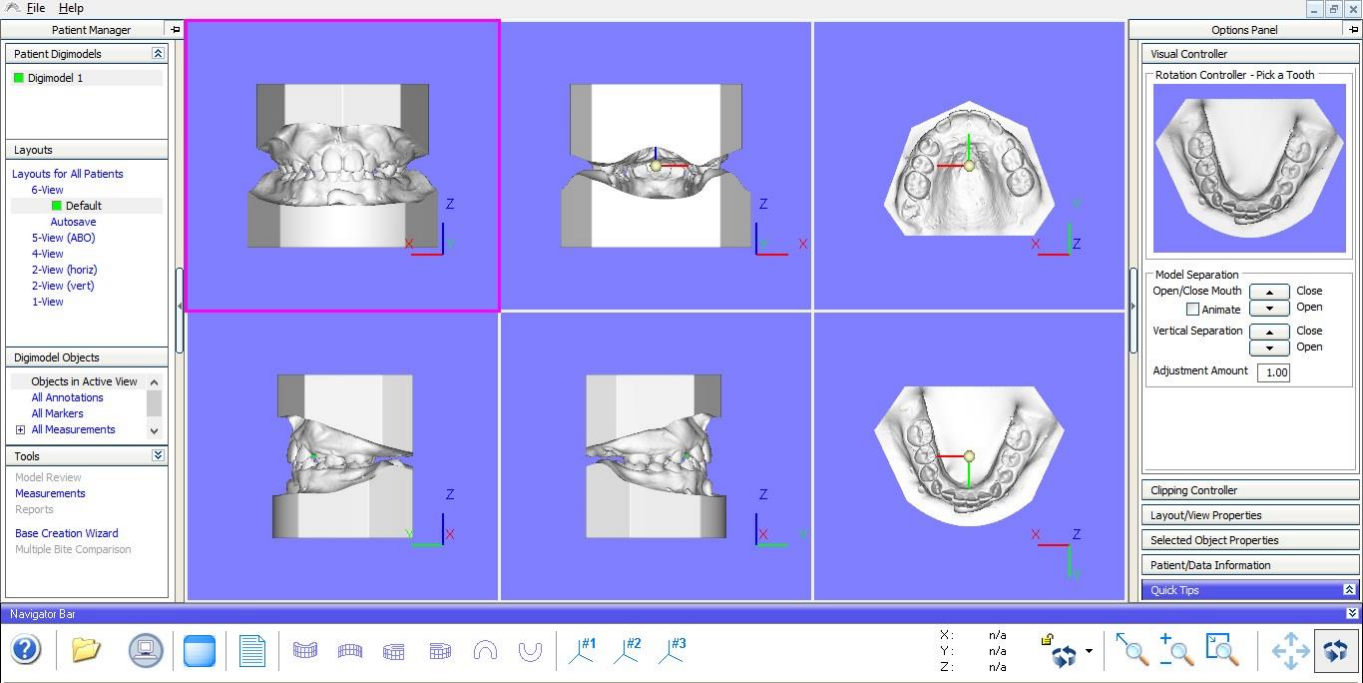
Digimodel® software program interface.

**Figure 2:**
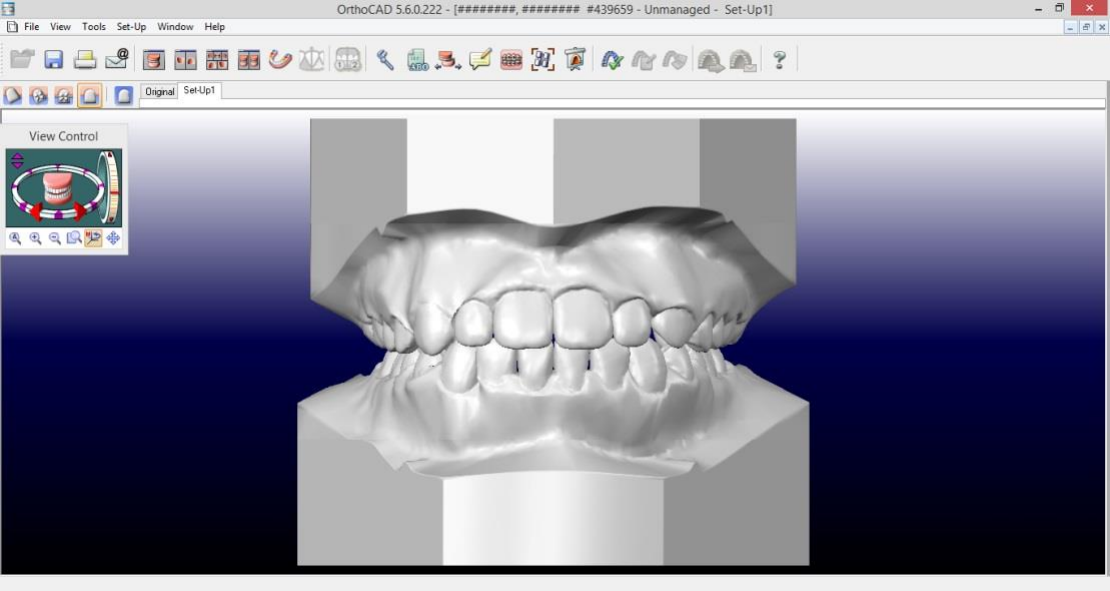
OrthoCAD® software program interface.

Both software programs allowed the visualization of the models in different axes in just one click through options present in the initial screen (Figures 1 and 2), which permitted and facilitated the task of classifying malocclusion.

**Figure 3:**
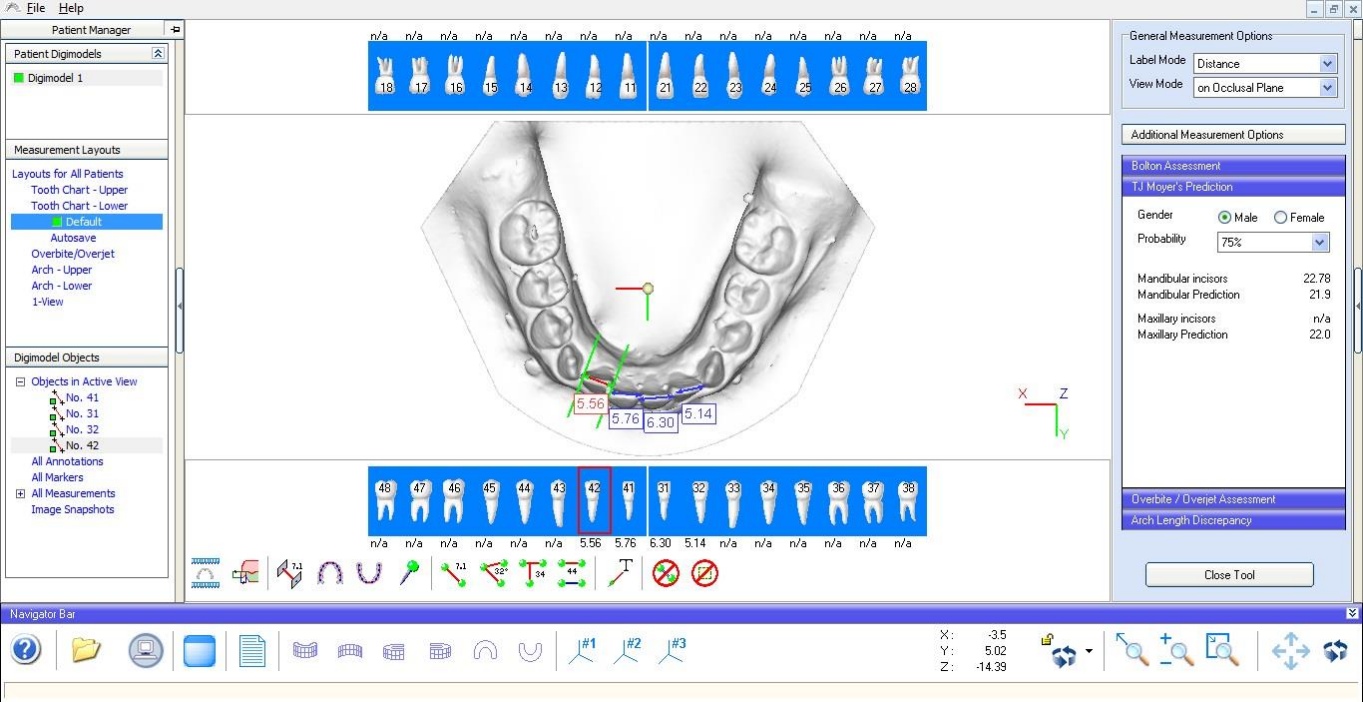
Tooth measurements taken in Digimodel® software program.

**Figure 4:**
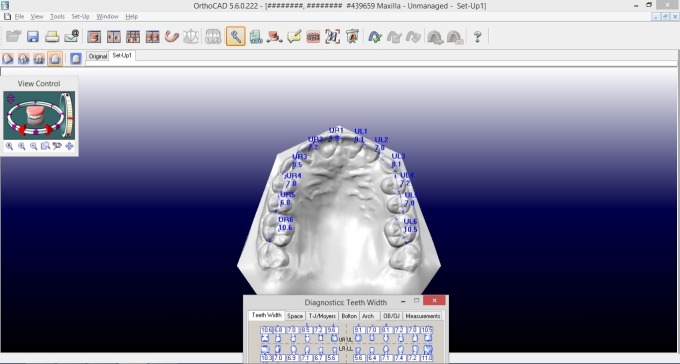
Tooth measurements taken in OrthoCAD® software program.

In models analysis, both software programs required tooth-to-tooth measurements and performed calculations of each analysis automatically, presenting them in a separate window (Figures 3 and 4).

## Discussion

The software progrsams Digimodel® and OrthoCAD® automate the calculations necessary to perform two important analyses for orthodontic diagnosis (bone‒dental discrepancy and inter-arch dental volume compatibility), as well as facilitate the classification of malocclusions through rapid access to different view axes/angles necessary for this task.

The accuracy, precision and reliability of Digimodel®^33,34^ and OrthoCAD®^35,36^ have been demonstrated in other studies. However, studies on the usability of this type of software program are still extremely scarce.^28^ In addition, the lack of standardization in the scientific term used to find articles on usability,^37^ as well as the unavailability of the Portuguese language in software, are barriers that may hinder advances in Brazilian orthodontics in this area, depriving many orthodontists who do not speak English of using this technology.

The concept of usability, brought by the ISONORM 9241, points to it as a measure that evaluates the ease with which a specific group of professionals can understand and perform a certain task in a computer program.^27^ In view of this, the usability of the OrthoCAD® software can be considered inferior to that of the Digimodel® software program due to its complexity in finding the tools (options) that allow actions common to the use of orthodontists.^3-6^ This reduced usability of OrthoCAD® software program compared to Digimodel® software program, which can be attributed to two factors: a) the amount of functionality offered generates more confusion during navigation in the software program, demotivating users to understand and use it;^38^ b) the marking of points to measure distances does not always obey the clicked site on the screen. This is consistent with Westerlund et al,^28^ who considered the usability of OrthoCAD® and three other software programs as weak. Digimodel® was not one of them. However, if it were included in these authors' paper, it would probably also be considered weak if evaluated by the same criteria that they used.

The methodology of the present work sought to evaluate free access versions of the most researched software programs in the literature in order to verify if their use could be wider, considering their ease of use and availability of necessary tools for orthodontic diagnosis. However, as found by Hassan et al,^16^ it was verified that the software programs evaluated in the present study allowed to work only with files in their own format. This means that the universal file format for orthodontic digital models (STL) is not downloadable in these software programs, making it possible, for the time being, to use them mainly in foreign countries due to patents in Digital Dentistry.

OrthoCAD® software program, for example, is available since 1999^17^ when it was launched in the market and is the most widely used orthodontic digital visualization and analysis software program in graduate programs in orthodontics in Canada and the United States.^32^ Although it has several potential benefits for its users,^12^ its free access version does not make its usufruct possible in locations that have scanning systems capable of producing only digital models in their standard format. The evaluated version of Digimodel® software program also had this same limitation.

Considering that the free access versions of the software programs evaluated meet the important criterion of being accessible in terms of cost,^25,26^ the factors discussed so far demonstrate that although they present several important functionalities for orthodontic diagnosis and treatment planning, they also have limitations when it comes to meet the demand of the Brazilian orthodontists who work with generic manufacturers' scanners, which are usually more accessible to them.

It should be remembered that digital models allow, in addition to the advantages related to the clinical workflow itself, the reduction of the need for physical space for model keeping, as it happens in the case of plaster models.^12^ Therefore, it would be advantageous for Brazilian orthodontists since the small physical space of their offices could be used for purposes other than archiving objects that occupy significant volumes in cabinets or shelves. This issue must also be considered from the ethical and legal points of view. Law cases against orthodontists have increased in numbers over time, both abroad^31^ and in Brazil.^39^

The Brazilian Code of Ethical Dentistry^40^ recommends that the professional must file patient documentation, which includes their models, for an indefinite period.^41^ On the other hand, from the legal point of view, it is also important that models be stored because they can be used in civil cases in which there is allegation of possible dental error.^42^ Under Brazilian civil law, according to Law 13.105, of March 2015, in its articles 369, 422 and 441, it is not impeditive to the digital documents (among them the models) as legal proofs, since it is provided from a suitable source.^43^ In addition, the digital format for orthodontic models, which make the measurement of teeth in the arches possible, allows them to be used for expert purposes if necessary, such as human identification, limited in some cases to examination of dental information of the probable victim.^44^ In this context, with the guarding of patient models becoming unavoidable, its digital version would make it easier to comply with such ethical issues and to protect dental professionals in possible conflicts with patients.

Although the present study has focused on issues related to diagnosis and treatment planning, it should be remembered that orthodontic treatment itself also brings with it undesirable side effects, such as tooth enamel wear and patient hygiene difficulties.^45,46^ Ways to control them, even in the details of each procedure, can help the clinician beware of possible litigation.

## Conclusion

The free access versions of the evaluated software programs present usability limitations related to the language and the supported file format. Although OrthoCAD® and Digimodel® present important functionalities for orthodontic diagnosis and treatment planning, the first one may have a barrier to learning or adoption by professionals in face of their variety of functions and difficulty in marking points, while the other cannot allow continuity in the workflow or data export.

## Acknowledgements

None.

## Authors’ contributions

WMMS and RFS contributed to the concept and design. MF contributed to data acquisition, analysis and interpretation. MMOL contributed to data interpretation and revision of its intellectual content. MGL contributed to data interpretation and revision of its intellectual content. All authors have read and approved the final manuscript.

## Ethical issues

Not applicable.
